# Comparing ABA, AAB, and ABC Renewal of Appetitive Pavlovian Conditioned Responding in Alcohol- and Sucrose-Trained Male Rats

**DOI:** 10.3389/fnbeh.2020.00005

**Published:** 2020-02-13

**Authors:** Shaun Yon-Seng Khoo, Joanna Marie Sciascia, Alexa Brown, Nadia Chaudhri

**Affiliations:** Center for Studies in Behavioural Neurobiology, Department of Psychology, Concordia University, Montreal, QC, Canada

**Keywords:** renewal, context, reinstatement, relapse, alcohol, sucrose, reward, Pavlovian conditioning

## Abstract

Conditioned responding can be renewed by re-exposure to the conditioning context following extinction in a different context (ABA renewal) or by removal from the extinction context (AAB or ABC renewal). ABA renewal is robust in Pavlovian and operant conditioning paradigms. However, fewer studies have investigated AAB and ABC renewal of appetitive conditioning, and those that did predominantly used operant conditioning tasks. Renewal has theoretical relevance for extinction and for exposure-based treatments for substance use disorders that aim to extinguish reactivity to drug-predictive cues. We therefore investigated ABA, AAB, and ABC renewal of Pavlovian conditioned responding to cues that predicted either alcohol or sucrose. Male, Long-Evans rats (Charles River) were exposed to either 15% ethanol (Study 1: “alcohol”) or 10% sucrose (Study 2: “sucrose”) in their home cages. Next, they were trained to discriminate between two auditory stimuli (white noise and clicker; 10 s) in conditioning chambers equipped with distinct olfactory, visual, and tactile contextual stimuli (context A). One conditioned stimulus (CS+) was paired with fluid delivery (0.2 ml/CS+; 3.2 ml/session; alcohol or sucrose in separate experiments), and the second CS (CS−) was not. In all sessions (conditioning, extinction, and test), each CS was presented 16 times/session on a variable-time 67-s schedule, and entries into the fluid port were recorded. CS+ port entries were then extinguished by withholding fluid delivery either in context A or in a second, different context (context B). Next, we assessed ABA, AAB, and ABC renewal in the absence of fluid delivery. During extinction, CS+ port entries were initially elevated in context A relative to context B. ABA renewal of CS+ port entries occurred in both alcohol- and sucrose-trained rats. ABC renewal approached statistical significance when data from both experiments were combined. No AAB renewal was observed, and, in fact, alcohol-trained rats showed AAB suppression. These results corroborate the reliability of ABA renewal and suggest that ABC renewal is a modest effect that may require greater statistical power to detect. From a treatment perspective, the lack of AAB renewal suggests that exposure-based treatments for substance use disorders might benefit from implementation in real-world, drug-use contexts.

## Introduction

Substance use disorders represent a global health crisis, and effective treatments are still needed (Degenhardt et al., 2013; Whiteford et al., 2013). One form of behavioral treatment involves repeated, systematic exposure to drug-predictive cues in the absence of drug use, which extinguishes cue-elicited conditioned reactivity (Bouton and Bolles, 1979; Bouton and King, 1983). Through extinction, exposure-based treatment aims to reduce the capacity of drug-predictive cues to impact relapse. However, gains from this approach may be transient and confined to the treatment context (Monti et al., 1993; Drummond and Glautier, 1994; Carter and Tiffany, 1999; Miller et al., 2001; Conklin and Tiffany, 2002; Bouton et al., 2012; Mellentin et al., 2017).

This idea comes from research that has identified processes that threaten long-term extinction. One such process, called “renewal,” refers to “the recovery of an extinguished conditioned response when testing occurs in a context different from that in which extinction treatment took place” (Polack et al., 2013). One of the first reports of renewal used a task in which a tone conditioned stimulus (CS) was paired with a shock unconditioned stimulus (US) in a specific context called “context A” (Bouton and Bolles, 1979). Conditioned responding to the CS was then extinguished in a different context (context B) by presenting the CS without shock. At test, responding to the CS without shock was renewed in context A. This experimental design is called “ABA renewal,” where sequential letters represent the conditioning, extinction, and test contexts. ABA renewal has been widely reported in aversive Pavlovian conditioning paradigms (Bouton and Bolles, 1979; Bouton and King, 1983; Ji and Maren, 2005; Fujiwara et al., 2012) and has important implications for the nature of extinction (Rescorla, 1993; Bouton et al., 2011). First, it suggests that extinction does not permanently erase the original CS–US association that was acquired during conditioning. Second, it suggests that extinction may produce a new, inhibitory CS–no US association that is specific to the extinction context. The latter hypothesis is based on studies showing that removal from the extinction context was sufficient to trigger renewal. In those studies, renewal was tested in a new context following training and extinction in either the same (AAB renewal) or different (ABC renewal) contexts (Gunther et al., 1998; Harris et al., 2000; Corcoran and Maren, 2004; Thomas et al., 2004).

The renewal effect has clear implications for exposure-based treatments for substance use disorders that are conducted in treatment settings bearing little resemblance to real-world, drug-use contexts. Indeed, research conducted in rodents using operant conditioning procedures has found reliable evidence of ABA renewal for drug (Crombag and Shaham, 2002; Fuchs et al., 2005; Diergaarde et al., 2008; Hamlin et al., 2008; Wing and Shoaib, 2008; Chaudhri et al., 2009; Marinelli et al., 2009; Bossert et al., 2012, 2019; Palombo et al., 2017) and non-drug reinforcers (Hamlin et al., 2006; Zironi et al., 2006; Marchant et al., 2009; Todd et al., 2012). Interestingly, AAB renewal of operant responding was not detected in two studies using drug reinforcers (Crombag and Shaham, 2002; Fuchs et al., 2005), whereas in studies using food pellets, AAB renewal was either absent (Nakajima et al., 2000) or detected but modest compared to ABA renewal (Bouton et al., 2011). Finally, ABC renewal of operant responding was observed for food pellets (Bouton et al., 2011) and liquid sucrose (Zironi et al., 2006), but not for ethanol (Zironi et al., 2006). Thus, while there is extensive support for ABA renewal, the evidence for AAB and ABC renewal of appetitive operant behavior is inconsistent or sparse.

In appetitive Pavlovian conditioning studies, ABA renewal is also a reliable effect (Chaudhri et al., 2008b; Anderson and Petrovich, 2015); however, AAB and ABC renewal are understudied. One laboratory reported ABC renewal using a discriminative conditioning task, and just as in operant conditioning, ABC renewal appeared to be a numerically smaller effect than ABA renewal (Campese and Delamater, 2013). AAB renewal of appetitive Pavlovian conditioned responding for food pellets was observed in one study (Bouton and Ricker, 1994), but another study failed to detect AAB renewal (Goddard, 1999). ABC and AAB renewal have also been observed following overexpectation (Rescorla, 2007) or using an autoshaping procedure (Rescorla, 2008). A summary of studies that tested ABA renewal in conjunction with AAB and/or ABC renewal is presented in Table 1.

**TABLE 1:** Studies that examined ABA renewal in conjunction with either AAB or ABC renewal of appetitive conditioning.

**Year**	**Author(s) and Journal**	**Subjects**	**CS**	**Reinforcer/US**	**Context modalities**	**ABA**	**AAB**	**ABC**
**Operant conditioning**								
2000	Nakajima et al., 2000 *Learn Motiv*	Male Wistar	Lever	Food pellet	V, T, A, Chamber size, Transport	Yes	No	Not tested
2002	Crombag and Shaham, 2002 *Behav Neurosci*	Male LE	Light	Heroin–cocaine	V, A	Yes	No	Not tested
2004	Bossert et al., 2004 *J Neurosci*	Male LE	Tone-light	Heroin	V, T, A Circadian	Yes	No	Not tested
2005	Fuchs et al., 2005 *Neuropsycho- pharmacology*	Male SD	No CS	Cocaine	V, T, A, O	Yes	No	Not tested
2006	Zironi et al., 2006 *Behav Brain Res*	Male LE	No CS	Alcohol or sucrose	V, T, O	Yes	Not tested	Yes sucrose; No alcohol
2011	Bouton et al., 2011 *Learn Behav*	Female Wistar	No CS	Food pellet	V, T, O	Yes	Yes	Yes
2012	Todd et al., 2012 *Learn Behav*	Female Wistar	No CS	Food pellet	V, T, O, Chamber size	Yes	Not tested	Yes
2013	Todd, 2013 *JEP: Anim Behav Process*	Female Wistar	No CS	Food pellet	V, T, O, A, Room	Yes	Yes	Yes
2015	Bouton and Schepers, 2015 *JEP: Anim Learn Cogn*	Female Wistar	No CS	Food pellet	V, T, O, A, Room	Yes	Not tested	Yes
2017	Schepers and Bouton, 2017 *Psychol Sci*	Female Wistar	No CS	Food or sucrose pellet	Interoceptive (satiated vs. hungry)	Yes	No	Not tested
2017	Trask et al., 2017 *J Neurosci*	Male Wistar	No CS	Sucrose pellet	V, T, O, Room	Yes	Not tested	Yes
**Pavlovian conditioning**								
1994	Bouton and Ricker, 1994 *Anim Learn Behav*	Male and Female Wistar	Tone-light	Food pellet	V, T, O	Not tested	Yes	Not tested
1999	Goddard, 1999 *Learn Motiv*	Female SD	No CS	Food pellet	V, T, O	Yes	No	Not tested
2013	Campese and Delamater, 2013 *Behav Brain Res*	Male and Female LE	Tone-light	Food pellet	V, T, Chamber size, Chamber shape, Room	Yes	Not tested	Yes
2007	Rescorla, 2007 *Anim Learn Behav*	Male SD	Noise and Light	Food pellet	T, O	Yes	Yes	Yes
2008	Rescorla, 2008 *Q J Exp Psychol*	Female Carneau Pigeon	Light	Grain	V	Yes	Yes	Yes

*Column 3 contains the subjects used (LE: Long-Evans; SD: Sprague Dawley). Column 6 contains the context modalities used (V: visual, T: tactile, O: olfactory, A: auditory).*

Given the theoretical and clinical implications of renewal, we sought to develop a comprehensive picture of the renewal of appetitive Pavlovian conditioned responding. Consequently, we investigated ABA, AAB, and ABC renewal in rats that were trained to associate a discrete, auditory CS (CS+) with either alcohol or sucrose delivery in separate experiments. A second, control CS (CS−) was also present during conditioning sessions but was not explicitly paired with fluid delivery. Using this task, we previously reported selective ABA renewal of responding to an alcohol-predictive CS+ (Chaudhri et al., 2008b) that was dependent on dopaminergic (Sciascia et al., 2014) and cholinergic neurotransmission (Lacroix et al., 2017). We also reported ABA renewal of responding to a CS+ that predicted sucrose (Chaudhri et al., 2008b) and a reduction of this effect by optogenetic activation of the infralimbic prefrontal cortex during the CS (Villaruel et al., 2018). Given that ABA renewal is a reliable phenomenon across learning paradigms, we predicted that a return to the conditioning context following extinction in a different context would selectively renew CS+ responding in both alcohol- and sucrose-trained rats. By comparison, we anticipated that AAB and ABC renewal would be detectable, but modest effects.

We examined ABA, AAB, and ABC renewal concurrently in separate studies using rats trained with either alcohol or sucrose. This experimental design enabled us to compare the extinction of CS+ responding in the same context as conditioning (context A) and in a different context (context B). We conducted these comparisons in order to inform theoretical explanations of the renewal effects that we observed.

## Materials and Methods

### Animals

Male, Long-Evans rats (220–240 g on arrival; *n* = 75) were obtained from Charles River Laboratories (Saint-Constant, QC, Canada). Upon arrival, rats were individually housed in polycarbonate home cages (44.5 cm × 25.8 cm × 21.7 cm) in a climate-controlled vivarium that was maintained on a 12-h light/dark cycle (lights on at 07:00). Behavioral procedures were conducted during the light cycle. Food (Charles River Rodent Diet, Saint-Hubert, QC, Canada) and water were always available in the home cage. Acclimation to the vivarium as well as regular weighing and handling occurred for 6 days before experiments began. The Animal Research Ethics Committee at Concordia University approved all procedures, which concurred with guidelines from the Canadian Council on Animal Care.

### Apparatus

Behavioral procedures were conducted in conditioning chambers (ENV-009A; 32.8 cm × 32.8 cm × 32.8 cm; Med Associates, Inc., St Albans, VT, United States) that were housed within custom-made, ventilated, sound-attenuating melamine cubicles (53.6 cm × 68.2 cm × 62.8 cm) located in a behavioral testing room that was distinct from the vivarium. The side walls of each chamber were made of stainless-steel panels, and the rear wall, ceiling, and front wall were made of clear acrylic glass. The floors were made of metal bars that extended from the rear wall to the front wall (ENV-009A-GF). A fluid receptacle (ENV-200R3AM) was located 2 cm above the floor, near the center of the right wall, and infrared sensors (ENV-254-CB) measured fluid port entries. Fluid was delivered into the receptacle via a 20-ml syringe that was mounted onto a pump (PHM-100, 3.33 RPM) located outside the sound-attenuating cubicle. A white house light (75 W, 100 mA, ENV-215M) was located near the ceiling on the left side of the chamber. The left wall also featured a white noise amplifier with cage speaker (ENV-225SM, calibrated to 8 dB above background, approximately 80–85 dB) and a clicker stimulus (ENV-135M, 75–80 dB). A computer running Med-PC IV controlled fluid delivery and auditory stimulus presentations and recorded port entries.

### Drugs and Solutions

A 15% (v/v) ethanol solution was prepared by diluting 95% ethanol in tap water. Sucrose was dissolved in tap water to obtain a final concentration of 10% (w/v). Lemon, almond, and cedar wood odors were prepared by suspending lemon oil (Cat#: W262528, CAS#: 8008-56-8, Sigma-Aldrich, Oakville, ON, Canada), benzaldehyde (Cat#: B6259, CAS#: 100-52-7, Sigma-Aldrich), and cedar wood oil (Cat#: W522406, CAS#: 68990-83-0, Sigma-Aldrich) in tap water (10% v/v), respectively.

### General Procedures

#### Home Cage Fluid Exposure

One week after arrival, rats (initial *n* = 37, final *n* = 36 with 1 rat dropped due to aggressive behavior) were acclimated to the taste and pharmacological effects of ethanol in the home cage using a 24-h, intermittent-access, two-bottle choice procedure that induces high levels of ethanol consumption in rats (Wise, 1973; Simms et al., 2008; Sparks et al., 2014). Rats had access to water via a 400-ml plastic bottle for 7 days/week. However, on Mondays, Wednesdays, and Fridays, a 100-ml graduated cylinder containing 15% ethanol (“alcohol”) was placed onto the lid of the home cage for 24-h, for a total of 23 sessions. Before each session, alcohol cylinders, water bottles, and rats were weighed, and 24-h later, alcohol cylinders and water bottles were reweighed to record consumption. To mitigate the effects of side preference on intake, the placement of alcohol and water on the left and right sides of the cage lid was alternated across sessions. Spillage was accounted for by subtracting alcohol and water lost from bottles that were placed on empty cages from consumption during the corresponding session.

One week after arrival, a separate group of rats (initial *n* = 38, final *n* = 33 with 3 rats dropped due to aggressive behavior and 2 rats dropped for self-injuries over grooming) received intermittent access to 10% sucrose (“sucrose”) in an identical manner. Two 24-h sessions of sucrose exposure separated by 24-h were conducted, because unlike ethanol, rats do not require extensive acclimation to sucrose.

Consumption of ethanol and sucrose solutions in the first and last sessions of this phase for each experiment is shown in Table 2.

**TABLE 2:** Home cage consumption of 15% ethanol or 10% sucrose.

		**ABA**	**AAB**	**ABC**
**15% ethanol**	**ml**			
	**First session**	1.03 ± 0.34	1.82 ± 0.91	1.04 ± 0.58
	**Final session**	12.78 ± 2.2*	14.61 ± 2.91*	12.87 ± 2.72*
	**g/kg**			
	**First session**	0.41 ± 0.13	0.7 ± 0.3	0.41 ± 0.23
	**Final session**	3.06 ± 0.56*	3.37 ± 0.65*	3.1 ± 0.64*
**10% sucrose**	**ml**			
	**First session**	68.4 ± 3.69	63.23 ± 3.32	53.55 ± 5.8
	**Final session**	81.02 ± 3.05*	78.72 ± 3.16*	75.15 ± 7.77*
	**g/kg**			
	**First session**	22.17 ± 1.17	20.61 ± 1.15	17.19 ± 1.83
	**Final session**	23.77 ± 0.89	23.47 ± 1.1*	21.87 ± 2.28*

Data are presented as means ± SEM. *p < 0.05 compared to the first session (paired *t*-test).

#### Habituation to Conditioning Chambers and Context Familiarization

Following home cage fluid exposure, rats were transported on a cart from the vivarium to the behavioral testing room and handled individually for 1 min. The next day, they were placed into a designated conditioning chamber within the behavioral testing room for 20 min, during which time the house light was illuminated and entries into the fluid port were recorded. Chambers were set up as one of three contexts. Context 1 consisted of a smooth acrylic glass floor, black walls, and lemon odor applied to the waste pan. Context 2 had a wire mesh floor, clear walls, and almond odor applied to the waste pan. Context 3 had a perforated metal floor, striped walls, and cedar wood odor applied to the waste pan. Chambers were set up as context 1 on the first day of habituation, context 2 on the second day, and context 3 on the third day. Context pre-exposure is standard practice in renewal protocols (Nakajima et al., 2000; Bouton et al., 2011; Campese and Delamater, 2013; Todd, 2013).

#### Pavlovian Discrimination Training

After habituation, rats received Pavlovian discrimination training in 19 daily (Monday–Friday) sessions each lasting approximately 1 h. Session onset was indicated by illumination of the house light 5 min after initiating the Med-PC program. In each session, rats received 16 presentations each of a 10-s white noise and a 10-s clicker (5 Hz) stimulus. One stimulus was designated as the CS+ and was paired with 0.2 ml of fluid delivered into the fluid port across 6 s, starting 4 s after CS+ onset. The second stimulus, the CS−, was not explicitly paired with fluid delivery. Each trial consisted of a 10-s pre-CS interval, a 10-s CS interval, and a 10-s post-CS interval. The intertrial interval (ITI) was 45, 60, or 90 s. The ITI did not include the pre-CS and post-CS intervals and was presented pseudorandomly with a mean ITI of 67.5 s. A total of 3.2 ml of fluid was delivered in each Pavlovian discrimination training session, and ports were checked to ensure that all the fluid was consumed.

For each rat, Pavlovian discrimination training occurred in a specific context, referred to hereafter as “context A.” Designation of the white noise or clicker as the CS+, as well as the context configuration that served as context A was based on creating matched groups according to ethanol intake averaged across the last 2 days of sessions of home cage ethanol exposure or sucrose intake averaged across both sessions.

#### Extinction and Renewal

Two days after the last Pavlovian discrimination training session, extinction was conducted across 12 daily 1-h sessions. The same Med-PC program used during Pavlovian discrimination training was used for extinction. The CS+ and CS− were presented as during Pavlovian discrimination training and syringe pumps were activated but did not contain syringes.

The day after the last extinction session, responding to the CS+ and CS− in the absence of fluid delivery was tested in either the renewal context or a comparison context. Test 1 was followed by five Pavlovian retraining sessions, nine re-extinction sessions, and a second test. Both tests were counterbalanced and followed a within-subjects design.

##### ABA renewal

Pavlovian discrimination training was conducted in context A, extinction in context B, and renewal in contexts A and B (ABA vs. ABB). The test in context B provided a within-subjects comparison context against which to assess renewal in context A (Crombag and Shaham, 2002).

##### AAB renewal

Pavlovian discrimination training and extinction both occurred in context A, followed by renewal tests in context B or context A (AAB vs. AAA). The AAB design evaluated the hypothesis that removal from the extinction context is sufficient to precipitate renewal (Bouton et al., 2012). The test in context A provided a within-subjects comparison context against which to assess renewal in context B (Fonteyne and Baeyens, 2011). Context B is sometimes referred to as a “novel” context in this design; however, rats were familiarized to this context in a single session before the start of Pavlovian discrimination training. Previous studies have observed AAB renewal in animals that were familiarized to context B (Bouton et al., 2011; Campese and Delamater, 2013; Todd, 2013).

##### ABC renewal

Pavlovian discrimination training was conducted in context A, extinction in context B, and renewal in context A and context C (ABA vs. ABC). As with AAB renewal, the ABC design evaluated if removal from the extinction context was sufficient for renewal. Rats were familiarized to context C in a single session before the start of Pavlovian discrimination training. In the ABC design, context B can serve as a comparison context against which to assess renewal in context C. However, we sought to compare renewal in context A and context C and used responding at the end of extinction in context B as a baseline against which to assess ABC renewal. ABA has been used as a comparison against ABC in both renewal and false memory tasks (Fonteyne and Baeyens, 2011; Bae et al., 2015).

Using these procedures, we examined ABA, AAB, and ABC renewal in separate experiments that were run concurrently. We conducted two sequential studies that included all three renewal experiments. In study 1, the CS+ was paired with 15% ethanol (“alcohol”), whereas in study 2, the CS+ was paired with 10% sucrose (“sucrose”). With the exception of home cage fluid exposure, both studies used identical behavioral training procedures and were conducted by the same researcher in the same set of conditioning chambers.

### Data Analysis

We recorded port entries during 10-s pre-CS, CS, and post-CS intervals, as well as during the variable ITI. Our primary variable of interest for assessing renewal was a difference score (Δ CS port entries), which was calculated by subtracting pre-CS port entries from port entries made during the corresponding CS. This variable accounted for individual differences in port entry behavior across rats and has been used previously in appetitive Pavlovian conditioning experiments (Campese and Delamater, 2013; Sciascia et al., 2014; Panayi and Killcross, 2018; Khoo et al., 2019).

All analyses were conducted using SPSS 24 (IBM, New York, NY, United States). Data were analyzed using *t*-tests and analysis of variance (ANOVA), as specified in Section “Results.” Greenhouse–Geisser corrections were applied to degrees of freedom following a significant Mauchly test of sphericity (all ε < 0.75). Results where *p* < 0.05 were considered statistically significant.

Discrimination between Δ CS+ and CS− port entries at the end of Pavlovian discrimination training was assessed using a paired *t*-test. ABA and AAB renewal were assessed using an ANOVA with cue (CS+ and CS−) and context (renewal context and comparison context) as within-subjects repeated measures. All *post hoc* comparisons were Bonferroni adjusted. For ABC renewal, we included an extinction baseline in the analysis to determine if responding at test in context C or context A was significantly different from extinction responding in context B. This extinction baseline was obtained by averaging data from the last session of extinction and re-extinction. Thus, the ANOVA for this experiment included cue (CS+ and CS−) and context (extinction in context B, test in context A, and test in context C) as within-subjects repeated measures. We also examined renewal with *t*-tests on data collapsed across both experiments. Finally, we compared extinction conducted in the Pavlovian discrimination training context (context A) with extinction conducted in a different context (context B; collapsed across ABA and ABC experiments) using a mixed-design ANOVA with cue and session as within-subjects repeated measures and context (context A and context B) as a between-subjects factor.

### Data Availability

The datasets generated and analyzed for this article can be found in figshare (Khoo et al., 2020).

## Results

### Pavlovian Conditioning for Alcohol and Sucrose

All rats acquired an appetitive Pavlovian response to a CS that predicted either alcohol or sucrose. There were no statistically significant differences across ABA, AAB, or ABC experiments in CS+ port entries at the end of Pavlovian discrimination training for alcohol- or sucrose-trained rats. For alcohol-trained rats, mean ± SEM Δ CS+ port entries in the final session of training were 28.75 ± 6.45, 22.33 ± 3.92, and 22.58 ± 7.93 for the ABA, AAB, and ABC experiments, respectively, and one-way ANOVA showed no significant difference between experiments [*F*(2,33) = 0.33, *p* = 0.72]. For sucrose-trained rats, mean ± SEM Δ CS+ port entries in the final session of training were 24.27 ± 5.61, 14.36 ± 2.57, and 17.36 ± 2.08, for the ABA, AAB, and ABC experiments, respectively. One-way ANOVA did not reveal any significant differences between experiments [*F*(2,30) = 1.826, *p* = 0.18].

### Renewal of Responding to an Alcohol-Predictive Cue

#### ABA Renewal (*n* = *12*)

**FIGURE 1:**
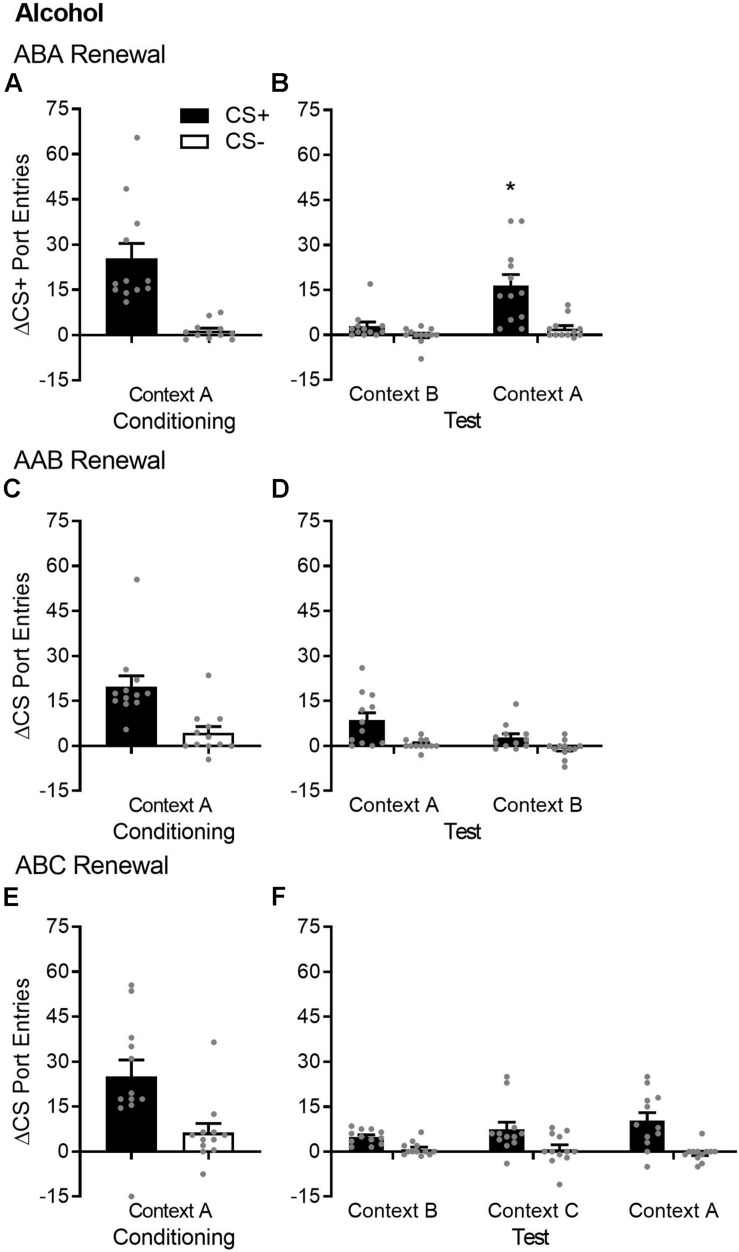
ABA but not AAB or ABC renewal of Pavlovian conditioned responding in alcohol-trained rats. **(A,C,E)** Average port entries during the CS+ (black bars) and CS– (white bars) across the final session of Pavlovian discrimination training and retraining in context A in the ABA, AAB, and ABC renewal experiments. In all cases, CS+ port entries were significantly higher than CS– port entries. **(B)** In the ABA renewal experiment, CS+ port entries at test in context A were significantly elevated compared to the control test in context B. **(D)** In the AAB renewal experiment, overall CS port entries were significantly lower at test in context B, relative to the control test in context A. **(F)** In the ABC renewal experiment, there was no significant difference in responding during either CS across an extinction baseline in context B (averaged across the final session of extinction and re-extinction) and renewal tests in context C or A. All data are mean ± SEM Δ CS port entries (CS minus pre-CS). *n* = 12 per experiment. Data from individual rats are overlaid as symbols on the bar graphs. *p < 0.05, Bonferroni-corrected *post hoc* comparison of CS+ port entries in context A versus context B.

By the end of Pavlovian discrimination training in context A, rats trained with alcohol significantly discriminated between the CS+ and CS− (Figure 1A). Port entries averaged across session 19 of Pavlovian discrimination training and session 5 of retraining were significantly higher during the CS+ than during the CS− [*t*(11) = 4.98, *p* < 0.001].

After extinction in context B, renewal was tested in the extinction context (ABB) and in the Pavlovian discrimination training context (ABA) (Figure 1B). Overall, port entries at test were higher in context A [context, *F*(1,11) = 10.13, *p* = 0.009] and during the CS+ [cue, *F*(1,11) = 19.19, *p* = 0.001]. However, there was a selective ABA renewal of CS+ port entries at test in context A [Cue × Context, *F*(1,11) = 9.19, *p* = 0.011]. Bonferroni-corrected *post hoc* comparisons showed that CS+ port entries were higher in context A compared to context B (*p* = 0.009), with no difference in CS− port entries as function of context (*p* = 0.075).

#### AAB Renewal (*n* = 12)

By the end of Pavlovian discrimination training in context A, rats significantly discriminated between the CS+ and CS− (Figure 1C). Port entries averaged across the final sessions of Pavlovian discrimination training and retraining were significantly higher during the CS+ than during the CS− [*t*(11) = 3.97, *p* = 0.002].

After extinction in context A, renewal was tested in the training/extinction context (AAA) and in a different context (AAB) (Figure 1D). Overall, port entries were higher during the CS+ than during the CS− [cue, *F*(1,11) = 16.42, *p* = 0.002] but were surprisingly lower at test in context B than in context A [context, *F*(1,11) = 10.02, *p* = 0.009]. There was no cue × context interaction [*F*(1,11) = 2.08, *p* = 0.18]. Thus, AAB renewal did not occur, and in fact, we observed AAB suppression, because CS responding was significantly lower in context B than in context A.

#### ABC Renewal (*n* = 12)

By the end of Pavlovian discrimination training in context A, rats significantly discriminated between the CS+ and CS− (Figure 1E). Port entries averaged across the final sessions of Pavlovian discrimination training and retraining were significantly higher during the CS+ than during the CS− [*t*(11) = 2.69, *p* = 0.021].

After extinction in context B, renewal was tested in a different context (ABC) and in the Pavlovian discrimination training context (ABA) (Figure 1F). To evaluate ABC and ABA renewal relative to baseline responding during extinction in context B, the ANOVA included data from both tests and from an extinction baseline. Overall, port entries were elevated during the CS+ compared to the CS− [cue, *F*(1,11) = 32.536, *p* < 0.001]. However, there was no main effect of context [*F*(1.34,14.68) = 0.52, *p* = 0.53, ε = 0.67] or cue × context interaction [*F*(1.19,13.04) = 2.45, *p* = 0.14]. Thus, according to this analysis, ABC renewal did not occur.

### Renewal of Responding to a Sucrose-Predictive Cue

#### ABA Renewal (*n* = 11)

**FIGURE 2:**
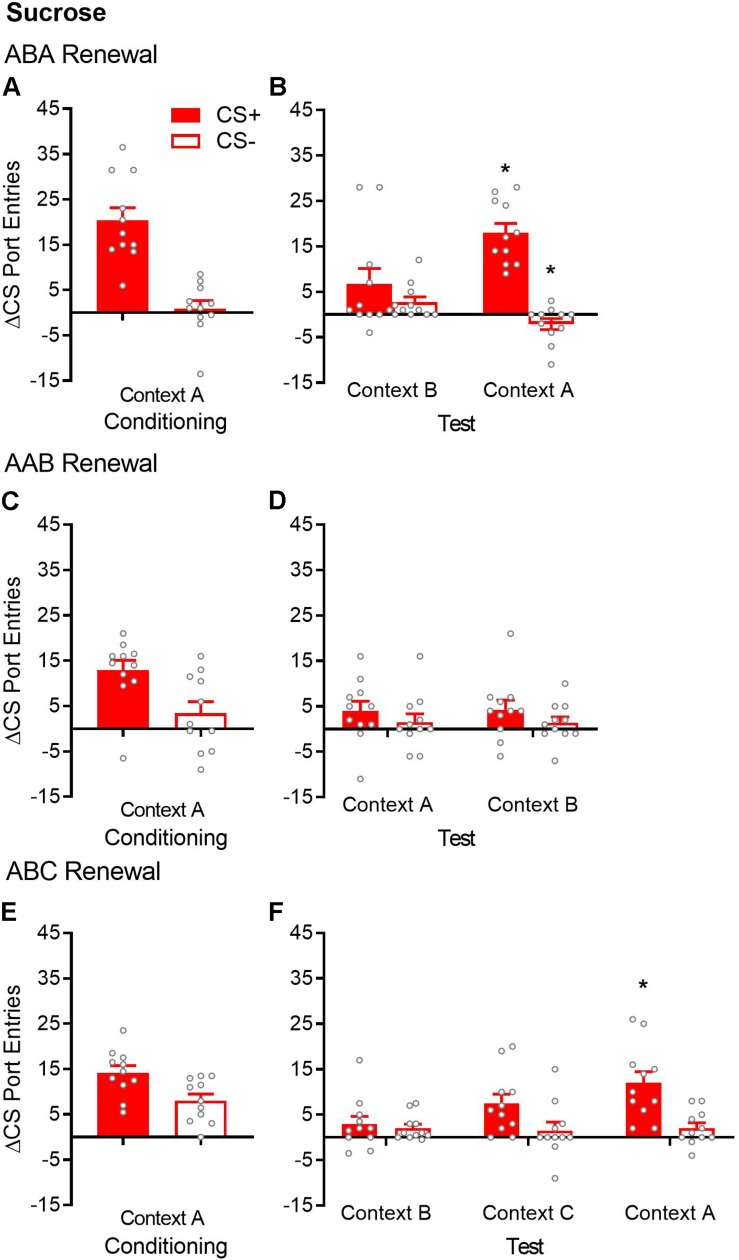
ABA but not AAB or ABC renewal of Pavlovian conditioned responding in sucrose-trained rats. **(A,C,E)** Average port entries during the CS+ (red bars) and CS– (white bars) across the final session of Pavlovian discrimination training and retraining in context A in the ABA, AAB, and ABC renewal experiments. In all cases, CS+ port entries were significantly higher than CS– port entries. **(B)** In the ABA renewal experiment, CS+ port entries at test in context A were significantly elevated compared to the control test in context B. **(D)** There was no evidence of AAB renewal. **(F)** In the ABC renewal experiment, CS+ port entries were significantly higher at test in context A relative to extinction in context B, indicative of ABA renewal. CS+ port entries at test in context C did not differ significantly from either extinction in context B or test in context A. All data are mean ± SEM Δ CS port entries (CS minus pre-CS). *n* = 11 per experiment. Data from individual rats are overlaid as symbols on the bar graphs. *p < 0.05 for a Bonferroni-corrected *post hoc* comparison between context A and context B.

By the end of Pavlovian discrimination training in context A, rats trained with sucrose significantly discriminated between the CS+ and CS− (Figure 2A). Port entries averaged across the last sessions of Pavlovian discrimination training and retraining were significantly higher during the CS+ than during the CS− [*t*(10) = 9.64, *p* < 0.001].

After extinction in context B, renewal was tested in the extinction context (ABB) and in the Pavlovian discrimination training context (ABA) (Figure 2B). Overall, rats responded more to the CS+ than to the CS− [cue, *F*(1,10) = 33.76, *p* < 0.001], with no significant main effect of context [*F*(1,10) = 2.27, *p* = 0.16]. However, ABA renewal was revealed by a significant cue × context interaction [*F*(1,10) = 22.13, *p* = 0.001]. Bonferroni-corrected *post hoc* tests showed that compared to those in context B, CS+ port entries were significantly higher in context A (*p* = 0.011), whereas CS− port entries were significantly lower (*p* = 0.004).

#### AAB Renewal (*n* = 11)

By the end of Pavlovian discrimination training in context A, rats significantly discriminated between the CS+ and CS− (Figure 2C). Port entries averaged across the final sessions of Pavlovian discrimination training and retraining were significantly higher during the CS+ than during the CS− [*t*(10) = 2.96, *p* = 0.014].

After extinction in the same context (context A), renewal was tested in the training/extinction context (AAA) and in a different context (AAB) (Figure 2D). ANOVA results indicated the absence of AAB renewal [context, *F*(1,10) = 0.0004, *p* = 0.984; cue, *F*(1,10) = 3.45, *p* = 0.093; cue × context, *F*(1,10) = 0.028, *p* = 0.87].

#### ABC Renewal (*n* = 11)

By the end of Pavlovian discrimination training in context A, rats significantly discriminated between the CS+ and CS− (Figure 2E). Port entries averaged across the last sessions of Pavlovian discrimination training and retraining were significantly higher during the CS+ than during the CS− [*t*(10) = 2.61, *p* = 0.026].

After extinction in context B, renewal was tested in a different context (ABC) and in the Pavlovian discrimination training context (ABA) (Figure 2F). Overall, rats continued to discriminate between the CS+ and CS− across extinction in context B and tests in contexts C and A [cue, *F*(1,10) = 9.37, *p* = 0.012]. Responding did not differ significantly across the three contexts, although the main effect showed a trend toward statistical significance [context, *F*(2,20) = 3.44, *p* = 0.052]. Interestingly, ANOVA revealed a significant cue × context interaction [*F*(2,20) = 5.37, *p* = 0.014]. Bonferroni-corrected *post hoc* tests supported ABA renewal, as CS+ port entries were significantly higher at test in context A, compared to extinction in context B (*p* = 0.034). However, CS+ port entries at test in context C did not differ from the extinction in context B (*p* = 0.20) or from that in context A (*p* = 0.23). These results support visual inspection of the data, which suggest that ABC renewal occurred in a subset of rats but was overall less consistent than ABA renewal.

### Renewal Effects on Data Collapsed Across Experiments

**FIGURE 3:**
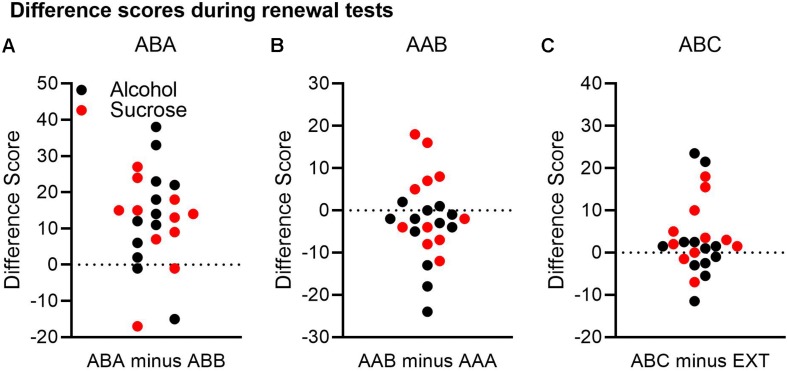
A comparison of renewal across both the alcohol and sucrose studies. A difference score was calculated for each renewal experiment by subtracting Δ CS port entries in the control context from Δ CS port entries in the renewal context. In this analysis, a positive difference score would support a renewal effect. **(A)** In the ABA renewal experiment, 19 of 23 rats had positive difference scores. **(B)** In the AAB experiment, 6 of 23 rats had positive difference scores, and 16 rats had negative difference scores. **(C)** In the ABC renewal experiment, 15 of 23 rats had positive difference scores, and 7 had negative differences scores.

The results presented above suggested that ABA renewal was the most reliable effect, AAB renewal was negligible, and ABC renewal was at best a modest effect. To explore these conclusions further, we calculated difference scores for each renewal experiment. For the ABA and AAB experiments, we subtracted Δ CS+ port entries at test in the comparison context from Δ CS+ port entries at test in the renewal context. For the ABC experiment, we subtracted Δ CS+ port entries during extinction in context B from Δ CS+ port entries at test in context C. Based on these calculations, a positive number indicated greater responding at test in the renewal context than in the comparison/extinction context. Figure 3 depicts these data collapsed across both experiments. To provide a measure of effect size, 95% confidence intervals of the difference are provided.

In the ABA renewal experiment (Figure 3A), 19 rats had positive difference scores, whereas four rats had negative difference scores. A *t*-test on the combined data from both the alcohol and sucrose experiments indicated that relative to the test in context B, responding was significantly higher in context A, confirming robust ABA renewal [*t*(22) = 4.51, *p* < 0.001, 95% CI = 6.74, 18.22].

In the AAB renewal experiment (Figure 3B), 6 rats had positive difference scores, 1 rat had a difference score of 0, and 16 rats had negative difference scores. A *t*-test on the combined data from both studies found no significant difference between the test in context A and the test in context B [*t*(22) = −1.46, *p* = 0.16, 95% CI = −6.41, 1.89].

Finally, in the ABC renewal experiment (Figure 3C), 15 rats had positive difference scores, while seven rats had negative difference scores and one rat had no difference. A *t*-test on the combined data indicated that the difference approached statistical significance [*t*(22) = 1.91, *p* = 0.069, 95% CI = −0.3, 7.3].

### Comparing Extinction in the Pavlovian Discrimination Training Context and in a Distinct Context

**FIGURE 4:**
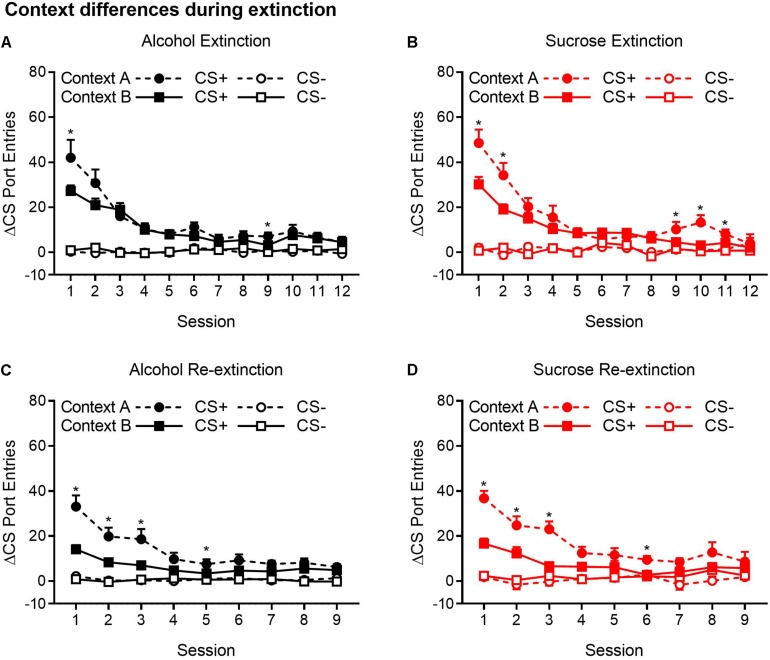
CS+ port entries were transiently elevated during extinction in context A compared to context B. Port entries across 12 extinction sessions prior to test 1 that occurred in **(A)** alcohol-trained rats (*n* = 12) and **(B)** sucrose-trained rats (*n* = 11). After the first renewal test, rats were retrained for 5 days and subjected to 9 days of re-extinction. Port entries are shown for **(C)** alcohol-trained and **(D)** sucrose-trained rats. All data are means ± SEM. *p < 0.05, Bonferroni-corrected *post hoc* comparisons for CS+ responding between the two extinction contexts.

Because ABA, AAB, and ABC renewal experiments were run concurrently, we compared extinction in the Pavlovian discrimination training context (context A) and in context B (Figures 4A–D). For both the alcohol and sucrose studies, data from the extinction phase in context B were collapsed across ABA and ABC experiments and compared to the extinction phase in context A from the AAB experiment. This analysis revealed that in both alcohol- and sucrose-trained rats, CS+ port entries were transiently but significantly elevated in context A than in context B at the start of the first extinction phase (Figures 4A,B), as well as the second extinction phase (Figures 4C,D) that occurred between tests. These statements are supported by statistical analyses that revealed a significant three-way interaction of cue × session × context for each condition (see Table 3 for all main effects and interactions).

**TABLE 3:** Statistical results of analysis of context-based differences in extinction.

	**Alcohol extinction (Figure 4A)**	**Sucrose extinction (Figure 4B)**	**Alcohol re-extinction (Figure 4C)**	**Sucrose re-extinction (Figure 4D)**
Cue	*F*(1,34) = 113.79	*F*(1,31) = 122.38	*F*(1,34) = 136.12	*F*(1,31) = 96.58
	*p* < 0.001*	*p* < 0.001*	*p* < 0.001*	*p* < 0.001*
Context	*F*(1,34) = 1.29	*F*(1,31) = 4.95	*F*(1,34) = 19.54	*F*(1,31) = 6.21
	*p* = 0.264	*p* = 0.034*	*p* < 0.001*	*p* = 0.018*
Session	*F*(4.97,169.02) = 31.15	*F*(6.29,194.89) = 33.19	*F*(4.74, 161.06) = 21.46	*F*(4.90,152.01) = 11.45
	*p* < 0.001*	*p* < 0.001*	*p* < 0.001*	*p* < 0.001*
	ε = 0.45	ε = 0.57	ε = 0.59	ε = 0.61
Cue × Context	*F*(1,34) = 2.76	*F*(1,31) = 5.29	*F*(1,34) = 18.03	*F*(1,31) = 23.60
	*p* = 0.11	*p* = 0.028*	*p* < 0.001*	*p* < 0.001*
Cue × Session	*F*(4.73,160.69) = 32.38	*F*(5.56,172.30) = 30.32	*F*(4.83,164.13) = 20.12	*F*(8,248) = 13.44
	*p* < 0.001*	*p* < 0.001*	*p* < 0.001*	*p* < 0.001
	ε = 0.43	ε = 0.50	ε = 0.60	
Session × Context	*F*(4.97,169.02) = 1.92	*F*(6.29,194.89) = 3.37	*F*(4.74,161.06) = 4.99	*F*(4.90,152.01) = 2.34
	*p* = 0.094	*p* = 0.003*	*p* < 0.001*	*p* = 0.046*
Cue × Session × Context	*F*(4.73,160.69) = 2.45	*F*(5.56,172.30) = 3.18	*F*(4.83,164.13) = 4.66	*F*(8,248) = 2.58
	*p* = 0.039*	*p* = 0.007*	*p* = 0.001*	*p* = 0.01*

*p < 0.05.

Figure 4A shows Δ CS port entries for the extinction phase that preceded the first renewal test in alcohol-trained rats. CS+ port entries were significantly elevated in context A relative to context B in extinction sessions 1 and 9. Similar results were obtained in sucrose-trained rats (Figure 4B), where CS+ port entries were significantly elevated in context A relative to context B in sessions 1–2 and 9–11.

In the nine sessions of re-extinction that occurred between tests, CS+ port entries in alcohol-trained rats (Figure 4C) were higher in context A than in context B in sessions 1–3 and in session 5. However, there was also a significant difference between contexts in CS− port entries in session 9. In sucrose-trained rats (Figure 4D), CS+ port entries were significantly higher in context A than in context B in sessions 1–3 and in session 6.

## Discussion

In the present experiments, we observed reliable ABA renewal of conditioned responding to a discrete, auditory CS that predicted either alcohol or sucrose. This effect was selective for the CS+ and did not occur for a CS− that was not explicitly paired with fluid delivery. In contrast, removal from a context associated with conditioning and extinction (AAB renewal) did not produce renewal in sucrose-trained rats, and in alcohol-trained rats it resulted in a surprising overall reduction in CS port entries. In both alcohol- and sucrose-trained rats, ABC renewal was not statistically significant, although for sucrose-trained rats, CS+ port entries at test in context C did not differ from CS+ port entries at test in either context A or context B. Finally, in a comparison of extinction, CS+ port entries were significantly higher in context A than in context B at the start of the extinction phase. The theoretical and clinical implications of these results, along with methodological considerations, are presented below.

### ABA Renewal

As predicted, we found evidence of ABA renewal in alcohol- and sucrose-trained rats. Across both experiments, the majority of rats responded more to the CS+ at test in context A, compared to context B (19 out of 23). These results are consistent with our prior research (Chaudhri et al., 2008b, 2013; Sciascia et al., 2014; Lacroix et al., 2017) and extend it to show that ABA renewal occurs using a within-subjects design. As before, ABA renewal was selective for the CS+, and a return to the conditioning context following extinction in a different context had no impact on CS− port entries. Overall, these findings concur with numerous published observations of ABA renewal in aversive and appetitive learning paradigms (Bouton and Bolles, 1979; Bouton and King, 1983; Bouton and Swartzentruber, 1991; Crombag et al., 2000; Tsiang and Janak, 2006; Zironi et al., 2006; Chaudhri et al., 2008b; Bouton et al., 2011; Valyear et al., 2017). They establish that our task parameters were sufficient to generate renewal and provide a basis for comparison with AAB and ABC renewal.

### AAB Renewal

Although AAB renewal has been reported in aversive Pavlovian conditioning studies (Bouton and Bolles, 1979; Tamai and Nakajima, 2000), we found no evidence of AAB renewal in either alcohol- or sucrose-trained rats. These results are consistent with two studies with drug reinforcers that failed to detect AAB renewal of operant conditioning (Crombag and Shaham, 2002; Fuchs et al., 2005). With a food pellet reinforcer, AAB renewal of operant conditioning was reported by one laboratory (Bouton et al., 2011; Todd et al., 2014), but not by another (Nakajima et al., 2000). A procedural difference between these studies is that the group that reported AAB renewal conducted magazine training in context B before the start of operant conditioning (Bouton et al., 2011; Todd et al., 2014). A context–reinforcer association formed during magazine training in context B may have influenced subsequent operant responding at test in context B.

In the present experiments, alcohol-trained rats in the AAB renewal experiment showed an overall reduction in CS port entries at test in context B, relative to context A, which is the opposite of a renewal effect. While this finding may be due to chance, we observed a similar effect in an unpublished AAB renewal experiment conducted using the shock-probe defensive burying task (Brown and Chaudhri, unpublished). This decrement was specific to CS port entries because there was no significant difference in ITI port entries at test for alcohol-trained rats [*t*(11) = −0.093, *p* = 0.928]. One explanation for this surprising result is a decrement in the generalization of conditioning (CS–US memory) across contexts. To mitigate such a decrement, we had familiarized rats to all three contexts before conditioning. However, the effects of this familiarization may have worn off by the time rats in the AAB renewal experiment were tested in context B. Thus, a switch to context B after training and extinction in context A might have had a non-associative effect on behavior which resulted in a reduction in CS port entries at test in context B.

Sucrose-trained rats did not show AAB renewal, but neither did they show a suppression of CS responding in context B relative to context A as was observed with alcohol-trained rats. Future studies are needed to replicate this difference across drug and non-drug reinforcers. Altogether, the present findings are consistent with studies that failed to detect AAB renewal of appetitive behavior (see Table 1).

### ABC Renewal

While in the AAB renewal design, extinction is conducted in the same context as conditioning, in the ABC renewal design conditioning, extinction and test all occur in different contexts. In both cases, renewal is tested in a context that differs from that of extinction; however, the ABC renewal design allows for the opportunity to learn that extinction occurs after a context switch.

We did not observe statistically significant ABC renewal in either the alcohol or sucrose experiment. In sucrose-trained rats, although the ANOVA revealed a statistically significant cue × context interaction, follow-up tests were inconclusive. Δ CS+ port entries in the ABC test (*M* = 7.5) were not significantly different from extinction in context B (*M* = 2.9) or the renewal test in context A (which was different from extinction with *M* = 12).

We considered the possibility that the effect size of ABC renewal may be smaller than ABA renewal and that by comparing ABA and ABC renewal, we might have reduced the statistical power that would have been available if we had compared an ABC test to an ABB test. To address this limitation, we collapsed data across both experiments and examined renewal using a difference score measure that subtracted Δ CS+ port entries in the comparison context from ΔCS+ port entries in the test context. For the ABC renewal experiment, we used the extinction baseline obtained in context B as the comparison context. The analysis for this experiment approached statistical significance, suggesting that there may be an ABC renewal effect that was smaller than we were able to statistically detect. Confidence intervals of effect size indicated that ABC renewal was approximately three to four times smaller than ABA renewal. These results therefore suggest that ABC renewal occurs in appetitive Pavlovian conditioning but that this effect is modest and may require greater statistical power to observe.

### Extinction Comparisons

When a switch from conditioning to extinction is accompanied by a change in context, there can be a decrement in conditioned responding that, as opposed to rapid extinction, reflects a lack of transfer of the original learning to the second context (Bouton, 2004). This “generalization decrement” is mitigated by exposure to both contexts prior to conditioning. In the present study, rats were familiarized to all three contexts before conditioning in separate, 20-min sessions. Magazine training was not conducted during these sessions, meaning that these contexts had no opportunity to become associated with sucrose or alcohol before Pavlovian discrimination training. Comparing across Figures 1A,C, 2A,C, 4A,C, there was no decrement in CS+ port entries triggered by a switch to context B, following conditioning in context A.

In both experiments, CS+ port entries in extinction were significantly higher in context A (the conditioning context) than in context B, and this effect occurred during the initial and second extinction phases. These results replicate prior operant conditioning research (Wing and Shoaib, 2008; Todd, 2013) and concur with our published data using a different Pavlovian conditioning procedure that equated exposure to contexts A and B before test (Remedios et al., 2014; Sparks et al., 2014; Millan et al., 2015; Sciascia et al., 2015; Valyear et al., 2018; Khoo et al., 2019).

The elevation in CS+ port entries during early extinction in context A versus context B may be related to context A gaining associative strength during Pavlovian discrimination training. This excitatory property of the context could summate with a CS–US association to energize CS+ port entries during extinction. The possibility that context A gained associative strength may have implications for AAB and ABC renewal. If rats in the AAB experiment experienced extinction of both context A and the CS+, then the resulting inhibitory memory may have been strong enough to prevent generalization of the CS–US memory to context B at test. It may also have contributed to the surprising and previously unreported AAB suppression effect observed in alcohol-trained rats. In contrast, if rats in the ABC renewal experiment only experienced extinction of the CS+, but not of the context–US association, then the inhibitory memory formed during extinction may not have been able to counter the generalization of the CS–US memory to context C at test, resulting in a weak renewal effect.

The similarity between the present data and prior operant conditioning results (Wing and Shoaib, 2008; Todd, 2013) showing a difference in extinction responding across contexts may also suggest that the magazine approach relies (at least in part) on instrumental contingencies. An instrumental contingency might arise from adventitious or superstitious conditioning of port entries that occur immediately before sucrose/alcohol delivery. Unlike port entries or operant responses like lever presses, other conditioned responses acquired during appetitive Pavlovian conditioning (e.g., head jerking) do not show context-based differences during extinction (Bouton and Peck, 1989), which also supports the idea that port entry responding might have an instrumental contingency. However, other studies provide compelling evidence that port entry behavior in appetitive Pavlovian paradigms is predominantly a Pavlovian conditioned response (Harris et al., 2013), and in at least one prior study, context-dependent differences in magazine approach were not observed (Carranza-Jasso et al., 2014). Thus, additional research is needed to delineate the contribution of Pavlovian and instrumental contingencies to port entry responding in appetitive Pavlovian paradigms.

### Theoretical, Methodological, and Clinical Considerations

There are a few psychological explanations for renewal that at first pass appear to be mutually exclusive but may ultimately occur in parallel with differential contributions to behavior based on task parameters. One explanation is that the extinction context may function as a negative occasion setter, such that release from this context will generate renewal (Bouton and Swartzentruber, 1986). This idea is supported by findings that extinguishing the conditioning context before test does not abolish renewal (Bouton et al., 2011; Todd et al., 2014) and that renewal is observed when the reinforcement histories of the conditioning and extinction contexts are equated (Todd, 2013). The extinction context may also function as a conditioned inhibitor, in which case removal from the extinction context would restore responding (Harris et al., 2000). However, context A may also acquire associative strength during conditioning, which could summate with residual associative strength of the CS following extinction to produce renewal. This account has been raised to explain why AAB and ABC renewal have often numerically weaker effects than ABA renewal (Polack et al., 2013).

In the present research, ABA renewal was more reliable than ABC renewal, and AAB renewal was not observed. The lack of AAB renewal might be attributed to differential levels of conditioning in this experiment, relative to the ABA and ABC renewal experiments. However, there were no statistically significant differences across experiment in CS+ port entries at the end of Pavlovian discrimination training, which suggests that the observed differences cannot be attributed to preexisting differences between experimental cohorts in baseline Pavlovian conditioning.

It is possible that using more than three elements in the configuration of contexts might have helped to make the contexts more discernibly distinct, increasing the possibility of detecting AAB renewal. In addition to having visual, olfactory, and tactile elements, we could have varied the shape of the conditioning box and the time of day at which conditioning/test and extinction were conducted. Conducting repeated conditioning, extinction, and test phases might also have increased discernibility across contexts, increasing the chances of detecting renewal.

A final methodological consideration of these experiments is that renewal was only tested in male rats. Other laboratories have reported ABA renewal of operant alcohol-seeking behavior in female rats (Bianchi et al., 2018) or inconsistent renewal of responding to a food-predictive CS in female rats (Anderson and Petrovich, 2015). However, it is notable that much of the historical literature on renewal has been conducted in female rats (Bouton and Peck, 1989; Bouton and Ricker, 1994; Goddard, 1999; Todd et al., 2012; Anderson and Petrovich, 2018a). The present results are consistent with this literature but cannot account for the possible impact of sex differences in appetitive learning that could differentially affect ABA, AAB, and ABC renewal.

Our data support the idea that if exposure-based treatment for substance use disorders occurs in a setting that is distinct from real-world, drug-use environments, then relapse facilitated by the renewal of conditioned responding to drug-predictive cues remains a possibility. Interestingly, the lack of AAB renewal in the present research supports the hypothesis that conducting exposure-based therapy in real-world, drug-use environments might prevent renewal-induced facilitation of relapse. One mechanism for this long-lasting effect may be through the extinction of both context–US and CS–US associations, which could produce a stronger inhibitory memory that does not allow the original CS–US memory to traverse contexts.

Finally, there is a burgeoning literature on the neural basis of ABA renewal in operant conditioning paradigms (Bossert et al., 2004; Fuchs et al., 2005; Chaudhri et al., 2008a; Crombag et al., 2008; Marchant et al., 2009; Marinelli et al., 2010). Fewer studies have examined the neural basis of renewal in appetitive Pavlovian learning paradigms (Chaudhri et al., 2013; Anderson and Petrovich, 2017; Anderson and Petrovich, 2018b; Villaruel et al., 2018), and only a handful of studies have directly compared neural processes underlying ABA, AAB, and ABC renewal. Trask et al. (2017) showed that pharmacological inactivation of the prelimbic cortex attenuated ABA renewal of operant responding for sucrose pellets; however, the same manipulation had no effect on ABC renewal in the same rats. In another study, Campese and Delamater (2013) found that pharmacological inactivation or lesions of the dorsal hippocampus had no effect on ABA or ABC renewal of Pavlovian responding to a cue that predicted food pellets (Campese and Delamater, 2013). Additional research is needed to directly investigate the neural basis of ABA renewal in conjunction with both AAB and ABC renewal designs.

## Conclusion

In addition to yielding theoretical insights about extinction, understanding the degree to which CS–US and CS–no US associations generalize across contexts may inform how to improve exposure-based treatment for substance use disorders. We observed ABA renewal of appetitive Pavlovian conditioned responding in alcohol- and sucrose-trained rats, which supports the reliability of this effect and suggests that a return to the conditioning context following extinction in a different context is a robust trigger for renewal. ABC renewal occurred in a subset of rats, but the analysis required a larger sample size to approach statistical significance. Combined with the lack of AAB renewal, our data suggest that removal from a context in which extinction was conducted is not a reliable trigger for renewal. Conducting extinction in the same context as conditioning, which produced transiently heightened CS+ port entries in the present research, might result in a stronger inhibitory memory that prevents AAB renewal and, for alcohol-trained rats, caused a surprising and previously unreported AAB suppression effect. These findings support a deep literature on the importance of context in behavioral responding and provide insight into how different approaches to extinction can influence later context-induced changes in behavior.

## Data Availability Statement

The datasets generated for this study are available on request to the corresponding author.

## Ethics Statement

The animal study was reviewed and approved by the Animal Research Ethics Committee, Concordia University.

## Author Contributions

NC and JS designed the experiments. JS conducted the experiments. SK analyzed and graphed the data. SK, AB, and NC wrote the manuscript. NC obtained funding and resources for the research and supervised JS, AB, and SK. All authors contributed to editing and finalizing the manuscript for publication.

## Conflict of Interest

The authors declare that the research was conducted in the absence of any commercial or financial relationships that could be construed as a potential conflict of interest.
